# Sap6, a secreted aspartyl proteinase, participates in maintenance the cell surface integrity of *Candida albicans*

**DOI:** 10.1186/1423-0127-20-101

**Published:** 2013-12-30

**Authors:** Leh-Miauh Buu, Yee-Chun Chen

**Affiliations:** 1 Department of Biotechnology, National Kaohsiung Normal University, No. 62, Shenzhong Rd., Yanchao District, Kaohsiung City 82444, Taiwan; 2 Division of Infectious Diseases, Department of Internal Medicine, National Taiwan University Hospital, Taipei, Taiwan; 3 Department of Medicine, National Taiwan University, Taipei, Taiwan

**Keywords:** Secreted aspartyl proteinases (Saps), Candidiasis, Cell surface integrity

## Abstract

**Background:**

The polymorphic species *Candida albicans* is the major cause of candidiasis in humans. The secreted aspartyl proteinases (Saps) of *C. albicans*, encoded by a family of 10 *SAP* genes, have been investigated as the virulent factors during candidiasis. However, the biological functions of most Sap proteins are still uncertain. In this study, we applied co-culture system of *C. albicans* and THP-1 human monocytes to explore the pathogenic roles and biological functions of Sap proteinases.

**Results:**

After 1 hr of co-culture of *C. albicans* strains and THP-1 human monocytes at 37°C, more than 60% of the THP-1-engulfed wild type and Δ*sap5 Candida* cells were developing long hyphae. However, about 50% of THP-1-engulfed Δ*sap6 Candida* cells were generating short hyphae, and more dead *Candida* cells were found in Δ*sap6* strain that was ingested by THP-1 cells (about 15% in Δ*sap6* strain vs. 2 ~ 2.5% in *SC5314* and Δ*sap5* strains). The immunofluorescence staining demonstrated that the Sap6 is the major hyphal tip located Sap protein under THP-1 phagocytosis. The *sap6*-deleted strains (Δ*sap6*, Δ*sap4/6*, and Δ*sap5/6*) appeared slower growth on Congo red containing solid medium at 25°C, and the growth defect was exacerbated when cultured at 37°C in Congo red or SDS containing medium. In addition, more proteins were secreted from Δ*sap6* strain and the β-mercaptoethanol (β-ME) extractable surface proteins from Δ*sap6* mutant were more abundant than that of extracted from wild type strain, which included the plasma membrane protein (Pma1p), the ER-chaperone protein (Kar2p), the protein transport-related protein (Arf1p), the cytoskeleton protein (Act1), and the mitochondrial outer membrane protein (porin 1). Moreover, the cell surface accessibility was increased in *sap6*-deleted strains.

**Conclusion:**

From these results, we speculated that the cell surface constitution of *C. albicans* Δ*sap6* strain was defect. This may cause the more accessible of β-ME to disulfide-bridged cell surface components and may weaken the resistance of Δ*sap6* strain encountering phagocytosis of THP-1 cells. Sap6 protein displays a significant function involving in maintenance the cell surface integrity.

## Background

*Candida* species are members of human normal microflora that reside in oral cavity, gastrointestinal tract, female genitalia and skin. Depending on the physiological status of the hosts, *Candida* species may convert from the commensally state to the pathogenic one and may cause disease from mucocutaneous superficial infection to systemic disseminated invasive candidiasis [1-3]. Among the members of genus *Candida*, the polymorphic species *Candida albicans* is the major contributor of candidiasis in humans [4,5]. For surviving under various physiological stress of human host, *C. albicans* could transform between yeast, pseudohyphae and true hyphae in response to the environmental change [6].

For many microorganisms, including *C. albicans*, cell wall is the first line to get in touch with host cells and also provides defense to against attacks from the host immune system. Besides, the cell wall components would be modified during morphogenetic programs to cope with the changes in environmental conditions [7-9]. Therefore, the cell wall plays important roles in maintenance the integrity and homeostasis of microorganisms. Cell wall proteins of *C. albicans* are in general highly mannosylated (mannoproteins) and enriched in the outer surface. They are attached mostly to short chains of β-1,6-glucan, to chitin via β-1,6-glucan, or directly to chitin, and to β-1,3-glucan in a lesser extent. Three types of covalently bound cell wall proteins in *C. albicans* have been described, including: the proteins bound to β-1,6-glucans through a glycosylphosphatidylinositol (GPI) moiety, the Pir (proteins with internal repeats) proteins attached to β-1,3-glucan by unknown alkali-sensitive bonds (possibly O-glycosidic linkages), and the proteins retained by disulfide bridges which can be extracted by treatment with reducing agents such as β-mercaptoethanol (β-ME) or dithiothreitol [10-13].

The model yeast *Saccharomyces cerevisiae* has been used extensively for study the fungal cell wall biogenesis and cell wall integrity. Numerous molecules have been identified to participate in cell wall construction and cell wall integrity signaling [14-16], including a group of yapsin family proteins. In *S. cerevisiae*, the members of yapsin family are five glycosylphosphatidylinositol (GPI)-linked aspartyl proteinases [17]. In *C. albicans*, ten secreted aspartyl proteinase (Sap 1 ~ 10) were identified and categorized to a Sap protein family [18-22], which have been investigated as the virulent factors during candidiasis [2,23,24]. Among the ten Sap proteins, Sap9 and Sap10 contain the C-terminal GPI-linked sequences which make them to be the yapsin homologues of *C. albicans*[22,25]. Studies have revealed that Sap9 and Sap10 are *C. albicans* cell surface-associated proteinases which cleave the covalently linked cell wall proteins [22,26] and involved their functions in maintenance the cell wall integrity and mediation the interaction between *C. albicans* with human epithelial cells and neutrophils [22,27].

In addition to *SAP9* and *SAP10*, expression of *SAP1* to *SAP6* have been extensively approached and demonstrated that *SAP1* ~ *3* were mainly expressed in yeast form *C. albicans* and *SAP4* ~ *6* were hypha-associated expression [2]. Many studies revealed that Sap proteins of *C. albicans* are virulent factors during candidiasis [2,23,24] but some studies reflect that Sap proteins are not essential for pathogenesis of *C. albicans*[28-30]. However, the precise biological functions of most Sap proteinases in *C. albicans* are still uncertain. In this study, we co-cultured the *C. albicnas* and THP-1 human monocytes to examine the hyphae development and escape behavior of different *sap*-null mutants when suffered phagocytosis. We demonstrated that Sap6 involved in the maintenance the cell surface integrity of *C. albicans*.

## Methods

### Strains and media

The *C. albicans* strains used in this study were listed in Table 1[22,26,31-35]. Strains were grown on/in YPD (1% yeast extract, 2% peptone, 2% glucose) complex medium. YP (1% yeast extract, 2% peptone) medium containing 0.1% glucose was used for induction of hyphae development. All media were added 40 mg of uridine per liter to minimize the effect of *URA3* gene [36]. *C. albicans* strains were cultured at 25°C to maintain the yeast form and incubated at 37°C for hyphae induction.

**Table 1:** ***C. albicans *** **strains used in this study**

**Strain type and no.**	**Genotype**	**Reference**
Clinical isolate SC5314	*URA3/URA3*	[31]
CAF4-2 (parental strain)	*ura3::imm434/ura3::imm434*	[32]
*sap2* (M12/BH52-1-17)	*sap2::hisG/sap2::hisG-URA3-hisG*	[33]
*sap5* (DSY452)	*sap5::hisG/sap5::hisG-URA3-hisG*	[34]
*sap6* (DSY346)	*sap6::hisG/sap6::hisG-URA3-hisG*	[34]
*sap4/5* (M28)	*sap4::hisG/sap4::hisG sap5::hisG/sap5::hisG-URA3-hisG*	[35]
*sap4/6* (M30)	*sap6::hisG/sap6::hisG sap4::hisG/sap4::hisG-URA3-hisG*	[34]
*sap5/6* (DSY437)	*sap6::hisG/sap6::hisG sap5::hisG/sap5::hisG-URA3-hisG*	[35]
*sap6* with pCIp10 (M1065)	*sap6::hisG/sap6::hisG* pCIp10	[35]
*sap6* with pCIp10-*SAP6* (M1067)	*sap6::hisG/sap6::hisG* pCIp10*-SAP6*	[35]
*sap9* (M1018)	*sap9::hisG/sap9::hisG-URA3-hisG*	[22,26]
*sap10* (M1171)	*sap10::hisG/sap10::hisG-URA3-hisG*	[22,26]

### Polyclonal antibody preparation

The construction and preparation of recombinant Sap proteins for generation of polyclonal antibodies have been described [37]. Because the highly conserved protein sequences between *C. albicans* and *S. cerevisiae*, we took advantage of several antibodies that were generated by using recombinant proteins of *S. cerevisiae* as antigens to recognize the homologues in *C. albicans*. The detailed properties of antibodies used in this study were listed in Table 2.

**Table 2:** Antibodies used in this study

**Antibody**	**Dilution**	**Property**	**Reference**
Anti-Sap6	1:5000	Polyclonal antibody, recognize *C. albicans* Sap4, Sap5, and Sap6 proteins.	[37]
Anti-Act1	1:5000	Polyclonal antibody, *S. cerevisiae* Actin as antigen, can recognize *C. albicans* Actin.	Dr. F-J S. Lee
Anti-porin 1	1:5000	Polyclonal antibody, *S. cerevisiae* porin 1 as antigen, can recognize *C. albicans* porin 1.	Dr. F-J S. Lee
Anti-Kar2	1:2000	Polyclonal antibody, Kar2 peptide of *S. cerevisiae* as antigen, can recognize *C. albicans* Kar2p.	Dr. F-J S. Lee
Anti-Pma1	1:5000	Polyclonal antibody, *S. cerevisiae* Pma1p as antigen, can recognize *C. albicans* Pma1p.	Dr. F-J S. Lee
Anti-Arf1	1:1000	Polyclonal antibody, *S. cerevisiae* Arf1p as antigen, can recognize *C. albicans* Arf1p.	Dr. F-J S. Lee

### *C. albicans* protein isolation and Western blot analysis

For total protein isolation, *Candida* cells were suspended in 200 μl of HEK solution (HEPES, pH7.4, 10 mM; EDTA 5 mM; KCl 50 mM) and added equal volume of glass beads. After a vigorous vortex for 10 min to break cells, 700 μl of HEK solution was added and incubated on ice for 10 min with occasional vortex. After centrifugation at 2,000 g for 5 min, the supernatant proteins were precipitated by 10% trichloroacetic acid (TCA). After centrifugation and washed with ddH_2_O, the protein pellet was suspended in 2× protein sample buffer and adjust pH value by 2 M Tris base, then incubated at 95°C for 10 min and stored at -20°C for further use. Western blotting was described previously [37].

### Extraction of cell wall associated components

*C. albicans* strains were cultured overnight in YPD medium at 25°C. Cells were harvested and transferred into YPD or YP medium containing 0.1% glucose, with initial density of OD_600_ = 1/ml, and cultured at 25°C (yeast form) or 37°C (hyphal form) for 3 hr with shaking. Cells were spun down, washed once with ddH_2_O, then suspended in extraction buffer (Tris–HCl, pH 8.8, 20 mM; KCl 50 mM; β-mercaptoethanol 1%, v/v). After rocking at 37°C for 30 min, cells were harvested and the medium components were precipitated by 10% TCA. The precipitate was harvested by centrifugation at 20,000 g, 4°C, for 10 min. The pellet of precipitate was washed once by ddH_2_O then suspended in 2× protein sample buffer and adjust pH value by 2 M Tris base. After incubation at 95°C for 10 min, the components were subjected to Western blot analysis [12,37].

### Co-culture of *C. albicans* with THP-1 human monocytes

The THP-1 human monocytic cell line [38] is maintained in RPMI1640 with 10% fetal bovine serum (RPMI-FBS) at 37°C in a humidified chamber containing 5% CO_2_. For co-culture, THP-1 cells were cultured in the 10-cm dishes for 2 days, then suspended cells in fresh RPMI-FBS and incubated at 37°C for 10 min before co-cultured with *Candida*. About 2×10^6^*Candida* cells were co-cultured with 2×10^5^ THP-1 cells in 1.5 ml of RPMI-FBS in a 2 ml microcentrifuge tube at 37°C incubator for indicated times with gentle rocking [38].

### Immunofluorescence staining of Sap proteins on hypha surface

*C. albicans* and THP-1 cells were co-cultured at 37°C for 30 min, then THP-1-engulfed *Candida* cells were harvested by low speed centrifugation and re-suspended in RPMI-FBS and incubated at 37°C for further 30 min. Co-cultured cells were harvested and suspended in PBS and loaded on poly-lysine coated cover glasses. The coated cells were fixed by 3.7% of formaldehyde in PBS for 15 min and were permeated by 0.2% TritonX100 for 3 min. After blocking, cells were incubated with anti-Sap6 antibody (1:800-dilution) for 90 min. The detailed procedure has been described [39].

### RNA preparation and reverse transcription-polymerase chain reaction

Total RNA of *Candida* cells was isolated by hot acid phenol method [40]. Before reverse transcription, 2 μg of total RNA was treated by DNaseI (Invitrogen). The cDNA was generated by SuperScriptIII (Invitrogen) with oligo-(dT)_12–18_ as primer. The expression of *SAP* genes was further identified by PCR using specific primers [35].

## Results

### The characteristics of engulfed *C. albicans* in THP-1 human monocytes

**Figure 1:**
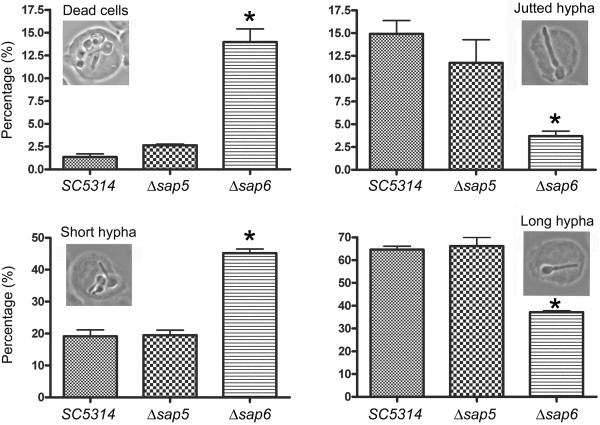
**Sap6 participates in the breakout process of *C. albicans *from phagocytosis of THP-1 monocytes.***Candida* cells and THP-1 cells were co-cultured at 37°C for 1 hr. Then cells were harvested and prepared for microscope inspection. Thirty views of microscope for each sample were subjected to dissect and calculate the growth characters of co-cultured *Candida* cells. *SC5314*: the wild type strain; Δ*sap5*: the *SAP5* gene deleted mutant; Δ*sap6*: the *SAP6* gene deleted mutant; short hypha: filament </= two mother cell length; long hypha: filament > two mother cell length. *: the difference between Δ*sap6* strain and wild type/Δ*sap5* strains is considered to be statistically significant (t test, P value < 0.01).

The hypha-associated expression of *SAP4-6* genes has been investigated as the potent virulent factors in mouse model of systemic candidiasis [34,41]. However, the biological functions of these Sap proteinases are still uncertain. The environment of systemic infection is too complicate to dissect protein functions of pathogens. Because macrophages may be the first encountered host defense cells during the invasive process of pathogens, we applied co-culture of *C. albicans* and THP-1 human monocytes to evaluate the pathogenic roles of Sap proteinases. After co-culture of *C. albicans* and THP-1 cells at 37°C for 1 hr, we inspected the status of *C. albicans* that engulfed by THP-1 cells. The microscopy showed that one or more *C. albicans* could be ingested by one THP-1 cell, and *C. albicans* could be induced to develop filamentous growth within THP-1 cells. The cell shape of THP-1 cells appeared extended by the elongated hyphae of ingested *Candida* cells. Some elongated hyphae could eventually burst the THP-1 cells that seemed to kill the monocytes, but some *Candida* cells would likely to be killed by monocytes that appeared as hollow or dense images that mostly stayed in the yeast- or germ tube-form within the THP-1 cells (Figure 1).

### Sap6 participates in the breakout process of *C. albicans* from phagocytosis

Because the Sap5 and Sap6 have been identified as the major expressed Sap proteins during hyphae development, we further characterized the properties of wild type strain SC5314, Δ*sap5* and Δ*sap6* mutants that were engulfed in THP-1 cells. We randomly took thirty views of each co-cultured samples and set several criteria to dissect the growth of *C. albicans* strains engulfed in THP-1 cells. The results revealed (Figure 1) that after 1 hr of co-cultur with THP-1 cells, most THP-1-engulfed SC5314 and Δ*sap5* cells were mostly developed long hyphae (about 60%). However, about 50% of THP-1-engulfed Δ*sap6* cells generated short hyphae, and more dead cells were found in THP-1-engulfed Δ*sap6* cells (about 15% in Δ*sap6* strain vs. 2 ~ 2.5% in SC5314 and Δ*sap5* strains). In addition, more protruding hyphae which pierced the THP-1 cells were found in THP-1-engulfed SC5314 and Δ*sap5* cells (about 12 ~ 15% in SC5314 and Δ*sap5* strains vs. 4% in Δ*sap6* strain). Hence, the Δ*sap6* mutant seemed with certain extent of defect in struggling of breakout from phagocytosis of THP-1 cells, although its hyphae formation is as efficient as other strains when cultured in the hypha-inducing media without THP-1 co-culture.

## Sap6 is distributed on hyphal tips of THP-1 engulfed *C. albicans*

**Figure 2:**
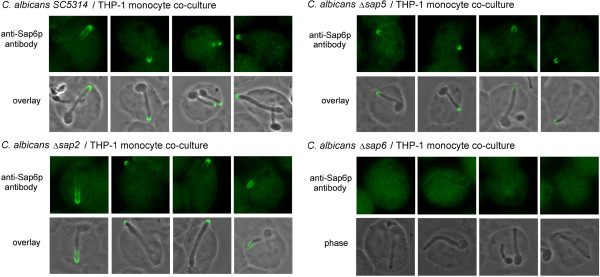
**Sap6 is distributed on hypha-tip of THP-1 engulfed *C. albicans*.** Co-cultured cells were harvested and subjected to immunofluorescence staining. Overlay: the fluorescence image was overlaid with the phase contrast image. *SC5314*: the wild type strain; Δ*sap2*: the *SAP2* gene deleted mutant; Δ*sap5*: the *SAP5* gene deleted mutant; Δ*sap6*: the *SAP6* gene deleted mutant.

Since the THP-1-engulfed Δ*sap6* cells exhibited slower hypha-extension, we further identified the expression of Sap proteins in *C. albicans* co-cultured with THP-1 monocytes by immunofluorescence staining using polyclonal anti-Sap6 antibody. The results demonstrated (Figure 2) that the Sap proteins could be detected on the hyphal distal-end of THP-1 engulfed *C. albicans*. Although the Sap5 is the most abundant secreted aspartyl proteinase in hypha-form *C. albicans*, the fluorescent signal still appeared on the hyphal distal-end of THP-1 ingested Δ*sap5* cells. However, fluorescent signal was vanished from the hyphal surface of THP-1 engulfed Δ*sap6* strain, suggesting that under phagocytosis the Sap6 is the main hyphal-tip located Sap protein.

### The Δ*sap6* strain displayed growth defect in media with cell wall attack components

**Figure 3:**
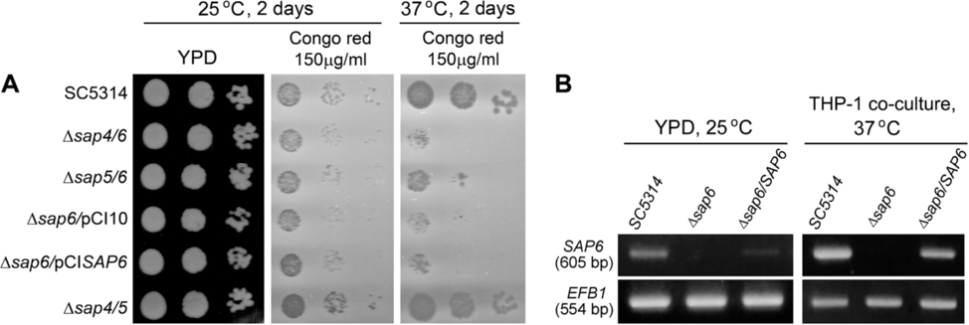
**The expression level of *SAP6 *is related to its function. (A)** The Δ*sap6* strains display growth defect in media with cell wall attack components. *C. albicans* strains were spotted on solid medium of YPD or YPD containing Congo red to verify the cell wall integrity of *Candida* cells. **(B)** Detection of *SAP6* expression by reverse transcription-PCR analysis. The YPD cultured or co-cultured cells were harvested after 30 min incubation at 25°C or 37°C, respectively. Total RNA was isolated and subjected to reverse transcription and PCR analysis.

Meanwhile, we found when picking the *C. albicans* cells from colonies by pipette tip, the Δ*sap6* mutant appeared more liquefied and clammy than other strains; hence we speculated that the cell surface characteristics of Δ*sap6* mutant may be different from wild type and other strains. Because the outer layer of *C. albicans* cells is cell wall, we examined the growth of *C. albicans* strains on solid media of YPD and YPD containing Congo red to verify the cell wall integrity of *Candida* cells. All tested strains demonstrated similar growth rate on YPD medium at 25°C (Figure 3A). However, *sap6*-null strains (Δ*sap6*, Δ*sap4/6*, and Δ*sap5/6*) appeared slower growth on Congo red containing medium at 25°C and displayed more severe growth defect when cultured at 37°C. The strain which contained one copy of re-integrated *SAP6* gene seemed to present a little rescue of growth defect on Congo red plate that cultured at 25°C but there was no observable effect at 37°C (Figure 3A). The mRNA expression level of re-integrated *SAP6* single-copy gene was approximately one-half of the wild type strain expression level in both yeast and hyphae form (Figure 3B), suggesting that the expression level of *SAP6* is important to fulfill its biological function.

**Figure 4:**
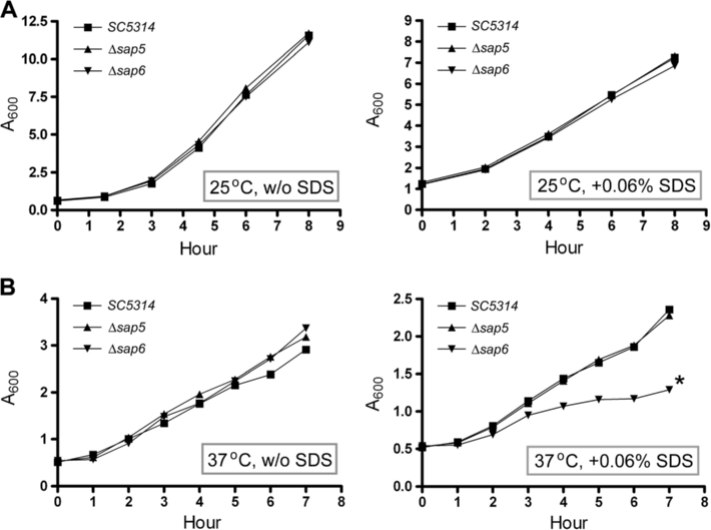
**The Δ*sap6 *strain is more sensitive to SDS under hypha-induced condition.***Candida* strains were cultured in rich medium or rich medium contained 0.06% SDS at 25°C **(A)** or 37°C **(B)**, respectively. Growth was measured at the specified time points. w/o: without. *: the difference between Δ*sap6* strain and wild type/Δ*sap5* strains is considered to be statistically significant (t test, P value < 0.05).

In addition, we cultured the *C. albicans* strains at 25°C in liquid rich medium or YPD rich medium containing 0.06% SDS (Figure 4A), the tested strains appeared almost the same growth rate in respective media. However, when the *C. albicans* strains were cultured at 37°C, the Δ*sap6* strain displayed evidently slower growth rate in medium containing 0.06% SDS (Figure 4B) and the single copy *SAP6* gene re-integrated strain did not displayed growth rescue (data not shown). This result is consistent with the Congo red plate assay which revealed the growth defect was more sever when *sap6*-null strains were cultured in media with cell wall attack components at 37°C. From these results, we suggested that the cell wall constitution may be deficient in Δ*sap6* strain.

### The accessibility of cell surface was increased in *sap6*-null strains

According to the cell wall constitution, wall components could be released or extracted by chemical agents. We applied the β-mercaptoethanol (β-ME) to extract surface proteins of *C. albicans* strains because this is a simple and efficient method to get a portion of outer layer proteins from *C. albicans*[10,13].

**Figure 5:**
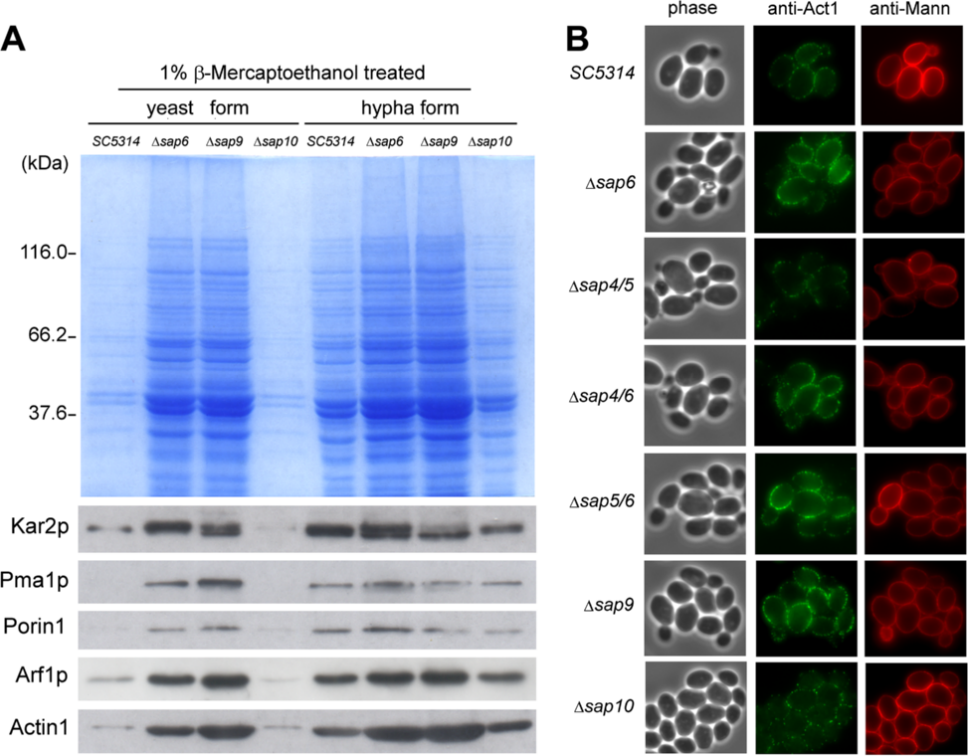
**The surface accessibility is increased in *sap6*-null strains. (A)** More proteins can be extracted by β-ME from Δ*sap6* and Δ*sap9* strains. The plasma membrane protein (Pma1p), ER-chaperone protein (Kar2p), protein transport-related protein (Arf1p), cytoskeleton protein (actin, Act1), and mitochondrial outer membrane protein (porin 1) were detected by specific polyclonal antibodies. **(B)** Immunofluorescence staining was applied to detect the actin without treating *Candida* cells into spheroplast. Strains with *sap6*-deletion (Δ*sap6*, Δ*sap4/6*, and Δ*sap5/6*) and Δ*sap9* mutant were more accessible to interact with Alexa Fluor™ 488 labeled anti-Act1 antibody. Anti-Mann: anti-mannan antibody.

The SDS-PAGE analysis revealed that in both yeast-form and hypha-form, the β-ME extractable proteins from Δ*sap6* mutant were evidently more than that extracted from wild type SC5314 strain (Figure 5A, upper panel). In addition, plenty of proteins also could be extracted by β-ME from a definite cell wall defect Δ*sap9* mutant [22], and the β-ME-extractable property of Δ*sap10* mutant was similar to wild type strain (Figure 5A, upper panel). The Western blotting demonstrated that plasma membrane protein (Pma1p), ER-chaperone protein (Kar2p), protein transport-related protein (Arf1p), cytoskeleton protein (actin, Act1), and trace of mitochondrial outer membrane protein (porin 1) could be detected in β-ME-extractable fraction, especially more abundant in the extracts of Δ*sap6* and Δ*sap9* strains (Figure 5A).

Since the actin could be extracted from cells by β-ME treatment and some actin filaments should close to the inner surface of plasma membrane, we applied immunofluorescence staining to detect the actin without treating *Candida* cells into spheroplast. The result showed that *sap6*-null strains (Δ*sap6*, Δ*sap4/6*, and Δ*sap5/6*) and Δ*sap9* strain were more accessible to interact with Alexa Fluor™ 488 labeled anti-Act1 antibody (Figure 5B), suggesting the surface accessibility was increased in *sap6*-null strains and Δ*sap9* strain.

Because the similar properties of Δ*sap6* and Δ*sap9* strains, we speculated that the cell surface constitution of Δ*sap6* strain was defect and gave rise to more accessible of β-ME to disulfide-bridged surface proteins; in addition, more proteins may be transported toward the cell surface to cope with the wall deficiency.

## Discussion

In this study, we attempted to explore the biological function of secreted aspartyl proteinases in *C. albicans*. Fortunately, in the co-culture system of *C. albicans* and THP-1 human monocytes, we found the discrepancy of hyphae development between Δ*sap6* strain and other tested strains during phagocytosis. Substantially, the Δ*efg1* mutant, which exhibited severe deficiency of filamentous growth in many cultivated conditions, could almost not generate hyphae within THP-1 cells (data not shown); and a moderate hypha-deficient mutant (Δ*cph1*) appeared delayed hyphae formation during phagocytosis (data not shown). In addition, the cell wall deficient Δ*sap9* strain [22] also exhibited poor hyphae development within THP-1 cells (data not shown). Therefore, co-culture of *C. albicans* and THP-1 human monocytes may apply as an efficient method for evaluation the potential factors relating to the invasiveness of *C. albicans* prior to examination by murine model.

A study using single and double null mutants of *sap* genes demonstrated that *C. albicans* strains, as long as with *sap6*-deletion, exhibited significantly reduced ability to invade and damage parenchymal organs in murine-model, although the hyphae development was normal and other Sap proteinases were still expressed [35]. Moreover, a study of murine keratitis also revealed that Sap6 is important for the pathogenesis of *C. albicans* keratitis [42]. In our co-culture system of *C. albicans* and THP-1 human monocytes, the protein level of secreted Sap5 was far more than the secreted Sap6 (Buu, unpublish), however, reduced capability of filamentous growth was found in Δ*sap6* strain of *C. albicans* within THP-1 monocytes. Besides, immunofluorescence staining revealed the Sap6 was the main Sap protein located on hyphal tips. These results highlight that the Sap6 may have distinct biological function involving in pathogenesis of *C. albicans*.

A class of GPI-anchored aspartyl proteinases known as fungal yapsins was firstly identified in *Saccharomyces cerevisiae*[25,43,44]. Yapsins in *S. cerevisiae* play important role in maintenance of cell wall integrity [17,25]. *C. albicans* has ten secreted aspartyl proteinases but only Sap9 and Sap10 have been characterized as the homologues of yapsins for they possessed the GPI-modification signal in their C-terminal peptide sequences [22,25]. Yps1, a plasma membrane reside yapsin of *S. cerevisiae*, has been investigated to be targeted to the vacuole and involving in the Golgi-associated proteolysis [45]. In addition, the *IFF* gene family of *C. albicans* encodes cell wall-related proteins. In this *IFF* gene family a secreted protein Iff11, which differs from other *IFF* family members in lacking a GPI anchor, has been investigated to play a role in cell wall organization, and presumed that the Iff11 may perform its function as it was transported through the protein secretory pathway [46]. The secreted Sap6 protein of *C. albicans* is also delivered through the conserved protein secretory pathway to the cell surface. Since the Sap6 is a proteinase, during transport through the secretory pathway it is possible that Sap6 provides cleavage-modification to maturate other co-delivered proteins which have main effect to establish the cell wall function.

In addition, *S. cerevisiae* Yps1 has been identified to cleave the extracellular inhibitory portion of the plasma membrane sensor protein Msb2p which is association with Sho1p for activation the Cdc42p-dependent MAPK pathway that controls filamentous growth and osmo-adaptive responses of *S. cerevisiae*[47]. In *C. albicans*, Msb2p is the sensor of defects in cell wall glycostructures and, associated with Sho1p, transmits the defective glycosylation signals to Cek1 MAP kinase pathway which functions in maintenance cell wall integrity [48]. Cleavage of Msb2p in *C. albicans* is also found to occur but is not performed by yapsin homologue Sap9 and Sap10, and Golgi-resident serine proteinase Kex2 is not involved as well [49]. A recent study revealed that Sap8 is a potent factor to be the major regulator of Msb2 processing in *C. albicans*[50]. These studies offer the hints that some physiological regulation processes are not exactly the same between *S. cerevisiae* and *C. albicans*, and there may have other unidentified cell surface-resident molecules also require further processing to exert their proper function when cells experience many different circumstances [51]. The Sap6 protein, though lacking a GPI-anchored moiety, may possibly retain on or associate with the cell surface constitution after it is secreted from the *C. albicans* and executes its proteinase activity on some surface molecules which may turn into active and participate in preservation the cell surface function.

## Conclusion

This study demonstrates that although Sap4, Sap5, and Sap6 have high identity in their DNA and protein sequences, Sap6 displays a significant function involving in maintenance the cell surface integrity. Hence, secreted Sap6 is able to be a member of cell surface-modifying enzymes. The precise molecular mechanism of Sap6 will be further characterized that may help us to realize the various functions of Sap proteinases.

## Abbreviations

SAP: Secreted aspartyl proteinase; GPI: Glycosylphosphatidylinositol; β-ME: β-mercaptoethanol; TCA: Trichloroacetic acid.

## Competing interests

The authors declare that they have no competing interests.

## Authors’ contributions

LMB designed and manipulated the experiments and wrote the manuscript. YCC discussed the experimental design and results with LMB and participated in manuscript writing. Both authors read and approve the final manuscript.
